# Novel real-time automation of combined frequency and low voltage substrate mapping to guide ablation for Brugada syndrome: a case report

**DOI:** 10.1093/ehjcr/ytae588

**Published:** 2024-10-29

**Authors:** Joseph Mayer, Jaffar Al-Sheikhli, Maria Niespialowska-Steuden, Elijah Behr, Tarvinder Dhanjal

**Affiliations:** Heart Rhythm Research Group, Division of Biomedical Sciences, Warwick Medical School, Clinical Sciences Research Laboratory, Clifford Bridge Road, Coventry CV2 2DX, UK; University Hospital of Coventry and Warwickshire NHS Trust, Clifford Bridge Road, Coventry CV2 2DX, UK; Heart Rhythm Research Group, Division of Biomedical Sciences, Warwick Medical School, Clinical Sciences Research Laboratory, Clifford Bridge Road, Coventry CV2 2DX, UK; University Hospital of Coventry and Warwickshire NHS Trust, Clifford Bridge Road, Coventry CV2 2DX, UK; University Hospital of Coventry and Warwickshire NHS Trust, Clifford Bridge Road, Coventry CV2 2DX, UK; Cardiovascular Clinical Academic Group, Molecular and Clinical Sciences Research Institute, St. George’s University of London, Cranmer Terrace, London SW17 0RE, UK; St. George’s University Hospitals NHS Foundation Trust, Cranmer Terrace, London SW17 0RE, UK; Heart Rhythm Research Group, Division of Biomedical Sciences, Warwick Medical School, Clinical Sciences Research Laboratory, Clifford Bridge Road, Coventry CV2 2DX, UK; University Hospital of Coventry and Warwickshire NHS Trust, Clifford Bridge Road, Coventry CV2 2DX, UK

**Keywords:** Brugada syndrome, Ventricular arrhythmias, Electro-anatomical mapping, Catheter ablation, Case report

## Abstract

**Background:**

Brugada syndrome (BrS) is an inherited cardiac condition that increases the risk of sudden cardiac death (SCD) due to ventricular arrhythmias. Catheter ablation has been shown to effectively reduce recurrent ventricular fibrillation (VF) episodes through targeting of abnormal electrograms predominantly located within the anterior surface of the right ventricular outflow tract. Signal frequency mapping is an emerging concept that provides further definition of pathological ventricular substrate.

**Case summary:**

A 66-year-old male with BrS was admitted to our institution with implantable cardioverter defibrillator shocks for VF. Electro-anatomical mapping (EAM) and ablation were performed utilizing a novel automated frequency-based strategy. Combined automated frequency and low voltage maps were generated to define high frequency, low voltage (HF-LVo) depolarization abnormalities within the QRS complex. Low frequency, low voltage (LF-LVo) regions from the QRS terminal notch to the T-wave offset were also identified. The combined HF-LVo and LF-LVo map areas totalled 12.4 cm^2^, compared to the conventional low voltage and late potential map areas, which were 44 cm^2^ and 27.8 cm^2^, respectively. The ablation strategy targeted HF-LVo and LF-LVo regions only. Following ablation, re-mapping demonstrated near complete abolition of HF-LVo and LF-LVo regions, with no inducible ventricular arrhythmias during extra-stimulation testing. During follow-up, ECG normalization was observed, with no further ventricular arrhythmias and a negative ajmaline challenge at 6 months.

**Discussion:**

Catheter ablation for BrS utilizing a novel automated combined frequency and low voltage EAM approach can objectively identify relevant substrate. The results demonstrate adequate substrate modification with comparable ablation target areas to previous studies and encouraging clinical outcomes.

**Learning points**

- Automated combined frequency and low voltage electro-anatomical mapping can objectively identify relevant substrate in Brugada syndrome.
- A combined frequency and low voltage catheter ablation approach can demonstrate favourable clinical outcomes.

## Introduction

Brugada syndrome (BrS) is an inherited cardiac condition that increases the risk of sudden cardiac death (SCD) due to the development of malignant ventricular arrhythmias, primarily ventricular fibrillation (VF).^1^ Currently, implantable cardioverter defibrillators (ICDs) are a proven effective treatment to prevent SCD in those with BrS.^2^ Implantable cardioverter defibrillators are currently recommended with a class I indication, as per the European Society of Cardiology (ESC) guidelines, for those with BrS who have previously survived a cardiac arrest or have sustained documented ventricular arrhythmias.^2^ Primary prevention implantation in patients with arrhythmic syncope remains a class IIa indication.^2^ However, ICDs do not treat the underlying substrate and the reported incidence of appropriate ICD therapies ranges from 10–50% during long-term follow-up.^3^

Catheter ablation has been shown to effectively reduce VF episodes by targeting abnormal electrograms (EGMs), predominantly located at the anterior epicardial surface of the right ventricular outflow tract (RVOT).^4,5^ Abnormal EGMs have been defined as low voltage, split or fractionated EGMs with multiple potentials and >20 ms isoelectric segments between peaks of individual components, and wide duration (>80 ms) or late potentials (LPs).^4^ Brugada *et al.* described an alternative but similar ablation strategy targeting fragmented, low frequency EGMs with an amplitude of <1.5 mV or associated wide duration (>80 ms), multiple (>3), or delayed components extending beyond the end of the QRS complex.^6^

Several groups have utilized these ablation targets and reported impressive long-term freedom from VF.^5,7^ More recently, two distinct types of abnormal EGMs based on the presence of high or low frequency components have been described and proposed to correlate with depolarization and repolarization abnormalities, respectively.^8^ High frequency potentials may be an equivalent to local abnormal ventricular activities and due to depolarization abnormalities from surviving cells in areas of fibrosis that can cause slow conduction and re-entry.^9^ Low frequency potentials may reflect dispersion of repolarization with recent evidence suggesting that repolarization abnormalities may be linked to delayed bipolar recordings.^10,11^

Signal frequency mapping is an emerging concept that provides further definition of pathological ventricular substrate.^12^ The Omnipolar Technology Near Field (OT-NF) feature of the EnSite™ X (Abbott, Minneapolis, MN) mapping system enables generation of automated frequency maps. To our knowledge, automated frequency maps utilizing the peak frequency (PF) algorithm to guide ablation for BrS has not been previously reported. Herein, we present a case utilizing novel automated combined frequency and voltage maps to guide catheter ablation.

## Summary figure

**Figure:**
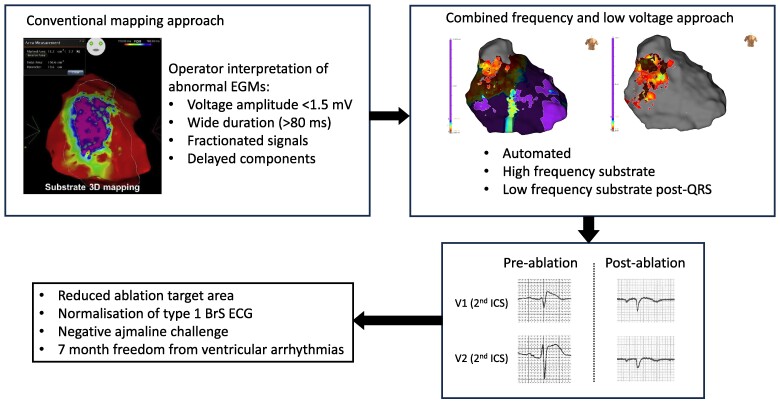


## Case presentation

**Figure 1:**
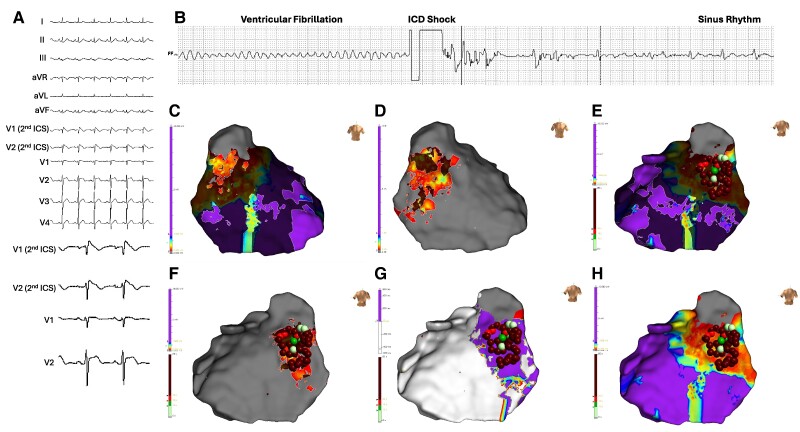
(*A*) A 66-year-old male with a 12-lead ECG demonstrating spontaneous Type I BrS ECG pattern. (*B*) Admission to our institution with VF episodes and ICD shocks confirmed with device (ICD) interrogation. Epicardial mapping and ablation performed utilizing a novel automated frequency-based strategy. (*C*) HF-LVo epicardial map displays frequency > 220 Hz. A discrete area of HF-LVo is located at the RVOT region. (*D*) LF-LVo epicardial map displays frequency < 160 Hz post-QRS, with a larger area of LF-LVo highlighted at the RVOT region. (*E*) Ablation lesions delivered at sites of HF-LVo on the visceral epicardial surface. (*F*) Ablation lesions delivered at sites of LF-LVo on the visceral surface. (*G*) Conventional LP map with ablation lesions on the visceral surface. (*H*) Conventional LVo substrate map with ablation lesions on the visceral surface (ICS: Intercostal Space).

A 66-year-old Caucasian male with a spontaneous Type I BrS phenotype, SCN5A gene variant negative and no other relevant past medical history, was admitted to our institution with electrical storm and five ICD shocks for VF (*Figure 1A* and *B*). On presentation, a physical examination was otherwise normal. The initial diagnosis of BrS was made in 2015 following an out-of-hospital cardiac arrest with subsequent ICD implantation. Cardiac MRI and coronary angiography revealed no evidence of cardiomyopathy, and the 12-lead ECG was diagnostic of spontaneous type 1 BrS characterized by J point elevation > 2 mV with coved ST elevation and T-wave inversion in the right precordial ECG leads, V1 and V2, positioned in the second intercostal space (*Figure 1A*). The patient had suffered 10 previous appropriate shocks prior to the index presentation which were managed without the use of drug therapy or catheter ablation. Pharmacological management with quinidine was not available due to limited supply. Quinidine has been shown to reduce shock frequency with a class IIa guideline recommendation in BrS patients who qualify for an ICD but have a contraindication, decline, or have recurrent ICD shocks.^2^ In keeping with a guideline-directed approach, catheter ablation was performed.

The procedure was performed under conscious sedation with epicardial access obtained utilizing the CO_2_ insufflation technique, and electro-anatomical mapping (EAM) performed using the Ensite X mapping system with the Advisor™ HD Grid mapping catheter, (Abbott, Minneapolis, MN) using EGMs filtered 30–300 Hz (*Figure 1C–H*). The pathological substrate in BrS is site specific predominantly the epicardial RVOT and for this reason, epicardial mapping is a guideline recommendation.^2,4^

**Figure 2:**
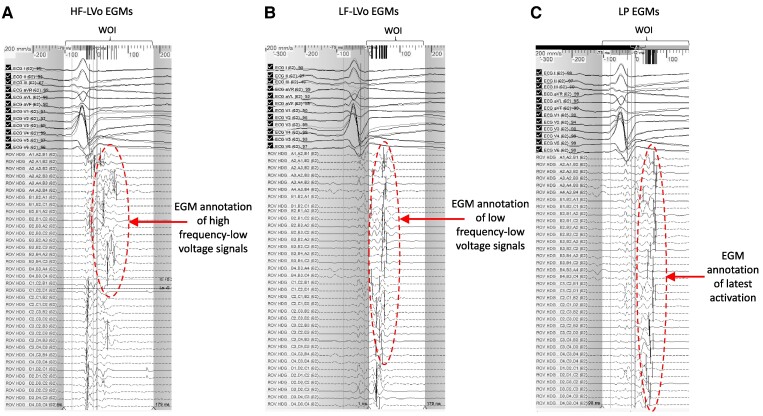
Window of interest (WOI) settings and example omnipolar EGMs to define high frequency-low voltage (HF-LVo), low frequency-low voltage (LF-LVo), and late potential (LP) maps. Vertical lines indicate EGM component annotated. (*A*) HF-LVo regions highlighting depolarization abnormalities with WOI from the QRS onset to T-wave. Encircled EGMs annotated as high frequency. (*B*) LF-LVo regions with the WOI set from the QRS terminal notch to the T-wave. Encircled EGMs annotated as low frequency. (*C*) Conventional late potential (LP) EGMs with corresponding signal annotation encircled.

The epicardial substrate map (6721 points used) confirmed a large low voltage (LVo) region on the anterior aspect of the RVOT (*Figure 1H*) with late potentials (LPs) (*Figure 1G*, representative EGMs shown in *Figure 2C*). LVo was defined as 1.5–0.2 mV based on background noise characterization (0.04–0.19 mV). Combined automated frequency and LVo maps (*Figure 1C* and *D*) were generated using the turbo-map function with the EGM window of interest (WOI) set to define:

1. High frequency-low voltage (HF-LVo) regions highlighting depolarization abnormalities incorporating the QRS onset to terminal portion of the T-wave (*Figure 1C*, representative EGMs shown in *Figure 2A*). High frequency was defined as >220 Hz. Within the LVo region, the mean PF was 264 Hz, compared to 200 Hz within the normal voltage region. The overall surface area of the HF-LVo region was 4.92 cm^2^.
2. Low frequency-low voltage (LF-LVo) regions with the WOI set from the QRS terminal notch to the T-wave offset (*Figure 1D*, representative EGMs shown in *Figure 2B*), with low frequency defined as <160 Hz. The surface area of the LF-LVo zone was 9.1 cm^2^, with a mean PF of 117 Hz.

The combined HF-LVo and LF-LVo map area was 12.4 cm^2^ compared to the conventional LVo substrate map area of 44 cm^2^ and LP (post-QRS) map area of 27.8 cm^2^.

**Figure 3:**
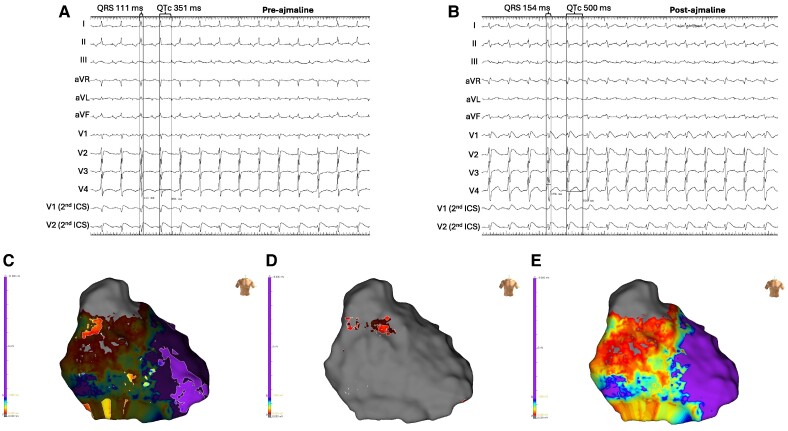
Electro-anatomical maps following ajmaline administration. (*A*) Twelve-lead ECG pre-ajmaline administration. (*B*) Twelve-lead ECG following ajmaline administration. Note prolongation of QRS width and QTc interval following ajmaline infusion. (*C*) Post-ajmaline HF-LVo map. (*D*) Post-ajmaline LF-LVo map. (*E*) Post-ajmaline conventional low voltage substrate map (1.5–0.2 mV).

A further epicardial substrate map was created following cautious administration of ajmaline (1 mg/kg over 10 min) limited by the degree of QRS widening and corrected QT (QTc) prolongation (*Figure 3*). The coved-type ST-segment elevation followed by a negative T-wave in lead V1 increased with an increase in both the QRS duration and QTc interval (*Figure 3A* and *B*). Abnormal EGMs became homogenized, with a reduction in the HF-LVo area to 1.93 cm^2^ and mean frequency reduction from 264 to 152 Hz (*Figure 3C*). The presence of LF-LVo regions were largely diminished (*Figure 3D*). The conventional LVo substrate area increased from 44 cm^2^ pre-ajmaline to 58.4 cm^2^ post-ajmaline (*Figure 3E*).

### Ablation strategy

Ablation targeted HF-LVo and LF-LVo regions only (*Figure 1E* and *F*) with an ablation endpoint of complete elimination of HF-LVo and LF-LVo substrate. Consistent with the BRAVO study, non-inducibility of sustained ventricular arrhythmias was not an ablation endpoint.^5^ Radiofrequency (RF) ablation at 30 W was delivered using the TactiFlex™ ablation catheter, sensor enabled™ (Abbott, Minneapolis, MN) with ablation parameters of either 2000 J or a 10 Ω impedance drop per lesion. The overall procedural time was 185 min, and total RF time was 31 min.

**Figure 4:**
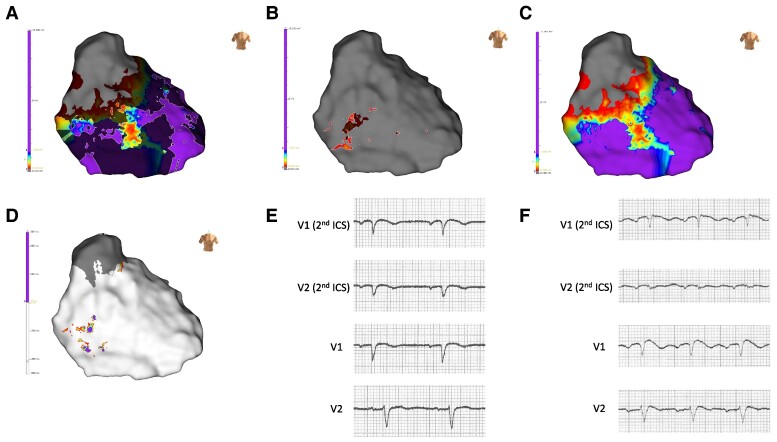
Epicardial re-maps post-ablation. (*A*) HF-LVo map. (*B*) LF-LVo map. (*C*) Conventional substrate map (1.5–0.2 mV). (*D*) Conventional LP map. (*E*) Three-month post-ablation right precordial ECG leads demonstrating normalization of spontaneous Type I BrS ECG pattern. (*F*) Six-month post-ablation right precordial ECG leads showing maximal response to ajmaline infusion.

Following ablation, re-mapping demonstrated near complete abolition of (*A*) HF-LVo and (*B*) LF-LVo regions as well as homogenization of (*C*) conventional substrate and (*D*) the more extensive LP region (*Figure 4*). Programmed ventricular stimulation with three extrastimuli from the RV apex and RVOT with the shortest coupling interval of 200 ms demonstrated no inducible ventricular arrhythmias. Intrapericardial triamcinolone (80 mg) was administered at the end of the procedure which reduces the risk of post-epicardial ablation pericarditis. The patient was discharged home the following day.

At 3-month follow-up, ECG normalization was observed (*Figure 4E*) and at 6-month follow-up, repeat ajmaline challenge was negative (*Figure 4F*) with ICD interrogation at 7 months confirming no further ventricular arrhythmias.

## Discussion

This is the first description of catheter ablation for BrS utilizing a novel automated combined frequency and low voltage EAM approach to identify epicardial abnormalities. The HF-LVo map defined fractionated components within the QRS and post-QRS region highlighting areas of abnormal depolarization, whereas the LF-LVo map defined low frequency abnormal signals that may reflect secondary repolarization abnormalities.^8,11^ Both high and low frequency components tend to be stable over time, supporting a primary conduction related pathophysiology. However, the low frequency signals may equally reflect more far field delayed depolarization waves.^13^ Nonetheless, this methodological approach utilizing novel mapping technology for BrS ablation allows the distinguishment of high frequency and low frequency omnipolar potentials with objective targeting of relevant substrate.

Previous conventional ablation approaches reported ablation target areas of 14 ± 7 cm^2^, comparable to the ablation target area in this case.^14^ The overall RF time of 31 min was similar to the recently reported BRAVO study (25 ± 16 min),^5^ suggesting adequate identification of abnormal EGMs utilizing this novel mapping strategy. Reassuringly, ECG normalization even post-ajmaline infusion was observed in this case, which has been reported as the only variable predictive of freedom from long-term VF recurrence.^5^

Large increases in low voltage substrate following administration of ajmaline have been previously reported resulting in more extensive ablation.^7^ A corresponding increase was also observed in this case; however, no increase in HF-LVo or LF-LVo regions were observed. This limited the area of ablation compared to that which would have been required if a more conventional approach was used. Ajmaline administration has been shown to not only increase the BrS substrate size but also cause marked increases in conduction delay and conduction block.^15^ Indeed, the diminished HF-LVo and LF-LVo regions observed following ajmaline infusion within our case may be due to conduction block in an already spontaneous type 1 patient.

This case report demonstrates that the utilization of automated frequency mapping to guide BrS ablation may be effective and could limit the amount of ablation required. The use of PF based ablation in BrS requires further validation and testing in prospective studies with longer follow-up to ensure that adequate substrate modification has been achieved.

## Data Availability

All data underlying this case report are available as part of the article. Additional data can be shared upon reasonable request to the corresponding author.

## Lead author biography

Joseph Mayer is a cardiac clinical scientist and member of the Heart Rhythm Research Group at the University of Warwick. The group have a special interest in heart rhythm abnormalities, with a particular focus on electro-anatomical mapping and ablation of ventricular arrhythmias.

## Notes

**Consent:** The authors confirm that written consent for submission and publication of this case report including images and associated text has been obtained from the patient in line with COPE guidance.

**Funding:** J.M.; National Institute for Health and Care Research [pre-doctoral fellowship/NIHR302718].

## References

[1] Brugada P, Brugada J. Right bundle branch block, persistent ST segment elevation and sudden cardiac death: a distinct clinical and electrocardiographic syndrome. A multicenter report. J Am Coll Cardiol 1992;20:1391–1396. 1309182 10.1016/0735-1097(92)90253-j

[2] Zeppenfeld K, Tfelt-Hansen J, de Riva M, Winkel BG, Behr ER, Blom NA, et al 2022 ESC guidelines for the management of patients with ventricular arrhythmias and the prevention of sudden cardiac death. Eur Heart J 2022;43:3997–4126. 36017572 10.1093/eurheartj/ehac262

[3] Dereci A, Yap SC, Schinkel AFL. Meta-analysis of clinical outcome after implantable cardioverter-defibrillator implantation in patients with Brugada syndrome. JACC Clin Electrophysiol 2019;5:141–148. 30784682 10.1016/j.jacep.2018.09.005

[4] Nademanee K, Veerakul G, Chandanamattha P, Chaothawee L, Ariyachaipanich A, Jirasirirojanakorn K, et al Prevention of ventricular fibrillation episodes in Brugada syndrome by catheter ablation over the anterior right ventricular outflow tract epicardium. Circulation 2011;123:1270–1279. 21403098 10.1161/CIRCULATIONAHA.110.972612

[5] Nademanee K, Chung FP, Sacher F, Nogami A, Nakagawa H, Jiang C, et al Long-term outcomes of Brugada substrate ablation: a report from BRAVO (Brugada Ablation of VF Substrate Ongoing Multicenter Registry). Circulation 2023;147:1568–1578. 36960730 10.1161/CIRCULATIONAHA.122.063367

[6] Brugada J, Pappone C, Berruezo A, Vicedomini G, Manguso F, Ciconte G, et al Brugada syndrome phenotype elimination by epicardial substrate ablation. Circ Arrhythm Electrophysiol 2015;8:1373–1381. 26291334 10.1161/CIRCEP.115.003220

[7] Pappone C, Brugada J, Vicedomini G, Ciconte G, Manguso F, Saviano M, et al Electrical substrate elimination in 135 consecutive patients with Brugada syndrome. Circ Arrhythm Electrophysiol 2017;10:e005053. 28500178 10.1161/CIRCEP.117.005053

[8] Pannone L, Monaco C, Sorgente A, Vergara P, Calburean PA, Gauthey A, et al High-density epicardial mapping in Brugada syndrome: depolarization and repolarization abnormalities. Heart Rhythm 2022;19:397–404. 34601129 10.1016/j.hrthm.2021.09.032

[9] Jais P, Maury P, Khairy P, Sacher F, Nault I, Komatsu Y, et al Elimination of local abnormal ventricular activities: a new end point for substrate modification in patients with scar-related ventricular tachycardia. Circulation 2012;125:2184–2196. 22492578 10.1161/CIRCULATIONAHA.111.043216

[10] Kurita T, Shimizu W, Inagaki M, Suyama K, Taguchi A, Satomi K, et al The electrophysiologic mechanism of ST-segment elevation in Brugada syndrome. J Am Coll Cardiol 2002;40:330–334. 12106940 10.1016/s0735-1097(02)01964-2

[11] Nagase S, Kataoka N, Morita H, Kamakura T, Ueoka A, Nakamura T, et al Demonstration of arrhythmia substrate-associated dispersion of repolarization by epicardial unipolar mapping in Brugada syndrome. JACC Clin Electrophysiol 2024;10:1576–1588. 38864810 10.1016/j.jacep.2024.05.012

[12] Payne JE, Woods C, Elshazly MB, Matthews A, Kroman A, Feng Z, et al A novel automated peak frequency annotation algorithm for identifying deceleration zones and ventricular tachycardia ablation sites. Heart Rhythm 2024;21:27–33. 37852563 10.1016/j.hrthm.2023.10.014

[13] Behr ER, Ben-Haim Y, Ackerman MJ, Krahn AD, Wilde AAM. Brugada syndrome and reduced right ventricular outflow tract conduction reserve: a final common pathway? Eur Heart J 2021;42:1073–1081. 33421051 10.1093/eurheartj/ehaa1051

[14] Grossi S, Bianchi F, Pintor C, Musumeci G, Gaita F. Transcatheter ablation in patients with Brugada syndrome. Eur Heart J Suppl 2023;25:C38–C43. 37125303 10.1093/eurheartjsupp/suad005 PMC10132615

[15] Nademanee K, Veerakul G, Nogami A, Lou Q, Hocini M, Coronel R, et al Mechanism of the effects of sodium channel blockade on the arrhythmogenic substrate of Brugada syndrome. Heart Rhythm 2022;19:407–416. 34742919 10.1016/j.hrthm.2021.10.031

